# Metabolic Profiling of Terpene Diversity and the Response of Prenylsynthase-Terpene Synthase Genes during Biotic and Abiotic Stresses in *Dendrobium catenatum*

**DOI:** 10.3390/ijms23126398

**Published:** 2022-06-07

**Authors:** Xinqiao Zhan, Yichun Qian, Bizeng Mao

**Affiliations:** 1 Institute of Biopharmaceuticals, Taizhou University, Taizhou 318000, China; 2 Institute of Biotechnology, Zhejiang University, Hangzhou 310000, China; 3170100649@zju.edu.cn; 3 Ministry of Agriculture Key Lab of Molecular Biology of Crop Pathogens and Insects, Hangzhou 310000, China; 4 Key Laboratory of Biology of Crop Pathogens and Insects of Zhejiang Province, Hangzhou 310000, China

**Keywords:** *Dendrobium*, terpene biosynthesis, metabolome, transcriptome, abiotic stress, biotic stress

## Abstract

*Dendrobium catenatum* is a widely cultivated Chinese orchid herb rich in abundant secondary metabolites, such as terpenes. However, terpene distribution and characterization of terpene biosynthesis-related genes remain unknown in *D*. *catenatum*. In this study, metabolic profiling was performed to analyze terpene distribution in the root, stem, leaf, and flower of *D*. *catenatum*. A total of 74 terpene compounds were identified and classified. Clustering analysis revealed that terpene compounds exhibited a tissue-specific accumulation, including monoterpenes in the flowers, sesquiterpenes in the stems, and triterpenes in the roots. Transcriptome analysis revealed that the ‘terpenoid backbone biosynthesis’ pathway was only significantly enriched in root vs. flower. The expression of terpene biosynthesis-related genes was spatiotemporal in the flowers. Prenylsynthase-terpene synthases (PS-TPSs) are the largest and core enzymes for generating terpene diversity. By systematic sequence analysis of six species, 318 *PS*-*TPSs* were classified into 10 groups and 51 *DcaPS*-*TPSs* were found in eight of them. Eighteen *DcaPS*-*TPSs* were regulated by circadian rhythm under drought stress. Most of the *DcaPS*-*TPSs* were influenced by cold stress and fungi infection. The cis-element of the majority of the *DcaPS*-*TPS* promoters was related to abiotic stress and plant development. Methyl jasmonate levels were significantly associated with *DcaTPSs* expression and terpene biosynthesis. These results provide insight into further functional investigation of *DcaPS*-*TPSs* and the regulation of terpene biosynthesis in *Dendrobium*.

## 1. Introduction

Terpenes are the largest and most diverse class of chemical substances that have been found in the plant kingdom, from moss to angiosperm [1]. Plants accumulate terpenes in multiple tissues that serve as defenses against diverse environmental stresses, pathogens, and herbivores [1,2]. Two spatially separated pathways exist for terpene biosynthesis in plants: the mevalonic acid (MVA), in cytosol and peroxisomes, and 2-C-methyl-D-erythritol 4-phosphate (MEP) pathways in plastids [3]. The MVA and MEP pathways are responsible for the formation of the isomeric five-carbon (isoprene) building blocks: isopentenyl pyrophosphate (IPP) and dimethylallyl pyrophosphate (DMAPP) [3,4]. These building blocks are further catalyzed to produce terpene biosynthesis precursors by prenylsynthases (PSs) and terpene synthases (TPSs), which play important roles in the structural diversity of plant terpenes [5].

Plant PSs are a class of enzymes responsible for the synthesis of isoprenoids, which contain three enzymes: geranylgeranyl pyrophosphate synthase (GGPS), farnesyl pyrophosphate synthase (FPS), and geranyl pyrophosphate synthase (GPS) [6]. The PSs are also core enzymes that participate in the synthesis of primary compounds such as chlorophylls, carotenoids, and derivatives including the hormones [6]. Plant TPSs can be divided into two classes based on the reaction mechanism and products formed [4,7]. Most known TPSs are in class I, which include the conserved DDxxD and the nonconserved NSE/DTE motifs. The class II activity resides in a separate domain ‘DXDD’ motif for the protonation-initiated cyclization of GGPP to copalyl pyrophosphate [4]. In addition, many TPSs have obvious N-terminal plastid transit peptides, ‘RRx8W’, which are essential for the catalysis of monoterpene cyclization [4]. The members of the TPS family are widely found in gymnosperms and angiosperms, but their distribution and function differ greatly. For instance, the number of putative *TPS* genes in seven species ranges from 1 in *Physcomitrella patens* to 69 in *Vitis vinifera* [4]. In Arabidopsis, phylogenetic analyses showed that 32 TPSs are classified into five subfamilies, some of which have been demonstrated to be mono- (C10), sesqui- (C15), di-TPS (C20), and sester-TPS (C25) derivatives [7]. *AtTPS* genes have distinct tissue-specificity and response mechanisms, such as *AtTPS21* in the stigma and sepals and *AtTPS03* in pollen [8], and *AtTPS02* and *AtTPS03* are induced locally at wound sites [9].

*D*. *catenatum* (also known as *D*. *officinale*) belongs to the Orchidaceae family and is a rare and precious Chinese herb. *D*. *catenatum* contains many bioactive extracts including polysaccharides, alkaloids, phenols, terpenes, coumarins, and flavonoids [10]. The genes involved in the biosynthetic pathways of alkaloids and flavonoids have been studied. P450 family genes, aminotransferase genes, and methyltransferase genes were induced by methyl-jasmonate (MeJA) and promoted sesquiterpene alkaloid biosynthesis [11]. *Flavanone* 3-*hydroxylase* (*F3H*) and *leucoanthocyanidin dioxygenase* (*LDOX*) affected the flux of dihydroflavonol and led to flavonoid accumulation in the stem [12]. However, terpene biosynthesis and the identification of related genes have rarely been reported in *D*. *catenatum*. In recent years, *D*. *catenatum* has been successfully cultivated and has become an important economic herb in China. Due to its antioxidant and antitumor activities, demand for secondary metabolites of *D*. *catenatum* is increasing across medicinal and therapeutic applications. To obtain a research basis for terpene metabolism in *Dendrobium* plants, we first identified the PS-TPS family in the *D*. *catenatum* genome and then made comprehensive assessments of the phylogenetic relationship, motif distribution, gene-specific expression, and responses to abiotic and biotic stresses. Our study provides insights into the evolution and function of PS-TPSs in medicinal *Dendrobium* plants.

## 2. Results

### 2.1. Composition and Classification of Terpene in Various Tissues of D. catenatum

**Figure 1:**
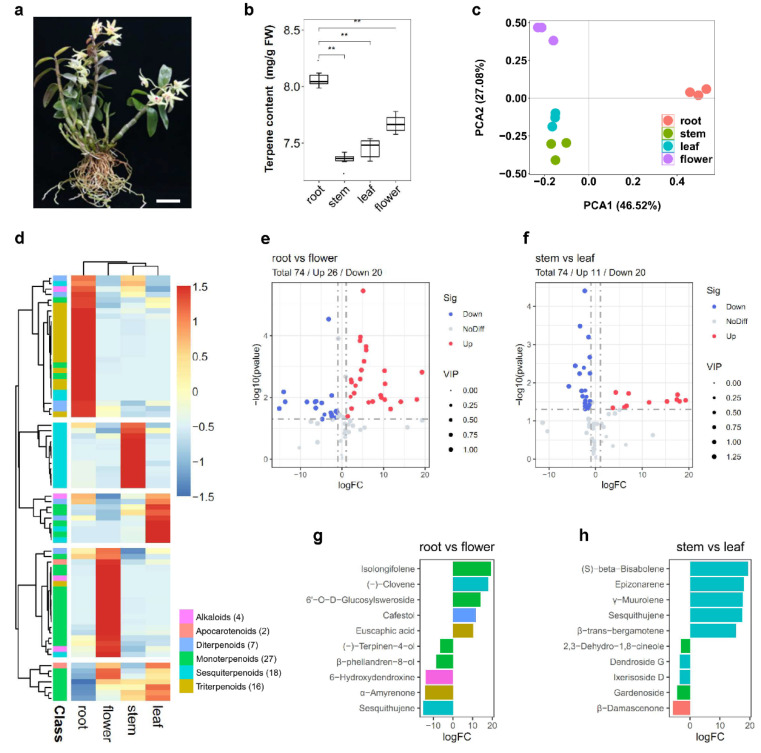
The distribution of terpenes in four tissues of *D*. *catenatum*. (**a**) Four tissues were used for terpene metabolic analysis. (**b**) The total terpene content in four tissues of *D*. *catenatum*. Values are means ± S.D. (*n* = 6). Student’s *t*-test, ** *p* < 0.01. (**c**) PCA analysis of volatile and non-volatile terpenes. (**d**) Terpene distribution in four tissues of *D*. *catenatum*. The color bar represents the normalization for log_2_-metabolite intensity using the Pheatmap software package. (**e**) Volcano plot showing the DAMs in root vs. flower. (**f**) Volcano plot showing the DAMs in stem vs. leaf. (**g**) Top ten DAMs in root vs. flower. (**h**) Top ten DAMs in stem vs. leaf.

The total terpene content was extracted and detected from fresh root, stem, leaf, and blooming flower of *D*. *catenatum* (Figure 1a). The terpene level of the root was significantly higher than that of other tissues (Figure 1b). To comprehensively analyze the distributions of terpenes in various tissues, gas chromatography-mass spectrometry (GC-MS) and liquid chromatography-tandem mass spectrometry (LC-MS) were performed to analyze the volatile terpenes and non-volatile terpenes, respectively. Principal component analysis (PCA) was used to analyze the first two characteristic components of the four tissue metabolites under non-supervision. Forty-one volatile terpenes and 33 non-volatile terpenes were separately formed into a cluster (Figure 1c). Of the 74 terpene metabolites identified, 2 were apocarotenoids, 4 were alkaloids, 27 were monoterpenes, 18 were sesquiterpenes, 7 were diterpenes, and 16 were triterpenes (Table S1). Clustering analyses revealed that most terpenes of *D*. *catenatum* exhibited a tissue-specific accumulation, with monoterpenes mainly in the flower and triterpenes in the root (Figure 1d). We further selected the differentially accumulated metabolites (DAMs) by a fold change of ≥2 or ≤0.5, and a variable importance in project (VIP) of ≥1 from root vs. flower and stem vs. leaf. Twenty-six DAMs were upregulated and twenty DAMs were downregulated in root vs. flower (Figure 1e and Table S2). Eleven DAMs were upregulated and twenty DAMs were downregulated in stem vs. flower (Figure 1f and Table S3). Furthermore, the 10 ten DAMs are shown in Figure 1g,h, and most of the terpenes were observed in four tissues, but several terpenes were found only in specific tissues such as α-amyrenone and 6-hydroxydendroxine in the flower, euscaphic acid in the root, γ-muurolene in stem, and dendroside G in the leaf.

### 2.2. Enrichment Analysis of Terpene Biosynthesis-Related Genes in Various Tissues of D. catenatum

**Figure 2:**
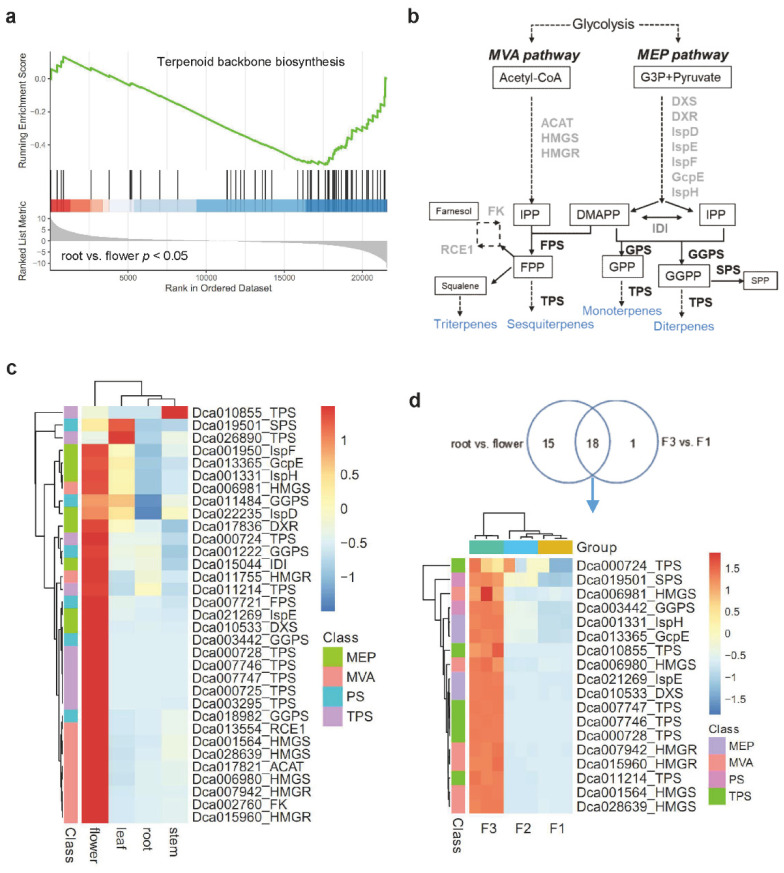
Enrichment analysis of terpene biosynthesis pathway in *D*. *catenatum* growth. (**a**) GSEA for root vs. flower comparison. Enrichment scores reveal the expression levels of enriched genes. *p* value < 0.05 is considered significant. (**b**) Overview of the two terpene biosynthetic pathways in plants. PS-TPSs are highlighted in black bold. (**c**) Expression profiles of terpene biosynthesis-related genes in four tissues. (**d**) Expression profiles of terpene biosynthesis-related genes during the three flowering phases. The color bar represents the normalization for log_2_-FPKM using the Pheatmap software package. Intermediates abbreviations are: DMAPP, Dimethylallyl pyrophosphate; DOXP, 1-deoxy-D-xylulose 5-phosphate; FPP, farnesyl pyrophosphate; GPP, geranyl pyrophosphate; GGPP, geranylgeranyl pyrophosphate; IPP, isopentenyl pyrophosphate; SPP, solanesyl pyrophosphate. Enzymes abbreviations are: AACT, acetoacetyl-CoA thiolase; DXR, DOXP reductoisomerase; DXS, DOXP synthase; FPS, FPP synthase; FK, farnesol kinase; GcpE, (E)-4-hydroxy-3-methylbut-2-enyl-pyrophosphate synthase; GPS, GPP synthase; GGPS, GGPP synthase; HMGR, HMG-CoA reductase; HMGS, HMG-CoA synthase; IDI, isopentenyl pyrophosphate isomerase; IspD, 2-C-methyl-D-erythritol 4-phosphate cytidylyltransferase; IspE, 4-diphosphocytidyl-2-C-methyl-D-erythritol kinase; IspF, 2-C-methyl-D-erythritol 2,4-cyclopyrophosphate synthase; IspH, 4-hydroxy-3-methylbut-2-en-1-yl pyrophosphate reductase; IspS, isoprene synthase; RCE1, prenyl protein peptidase; SPS, SPP synthase; TPS, terpene synthase.

To explore the expression of terpene biosynthesis-related genes in *D*. *catenatum* tissues, gene set enrichment analysis (GSEA) was performed on transcriptome data from four tissues. The KEGG pathway ‘terpenoid backbone biosynthesis’ was only significantly (*p* < 0.05) enriched in root vs. flower (Figure 2a). Terpene biosynthesis precursors, IPP and DMAPP, were produced by the plastidial MEP pathway and the cytosolic MVA pathway. IPP and DMAPP were then modified by PS-TPSs to generate terpene diversity (Figure 2b). A total of 33 enriched genes were associated with terpene biosynthesis (Table S4), and the encoded enzymes are labeled in Figure 2b. Among them, 28 genes showed the highest expression levels in the flowers (Figure 2c). However, one TPS gene (*Dca010855*) was highly expressed in the stem. Two genes, *TPS* (*Dca026890*) and *SPS* (*Dca019501*, *solanesyl pyrophosphate synthase*), were predominantly expressed in the leaf. We further explored the expression of terpene biosynthesis-related genes in early flower buds (F1), medium flower buds (F2), and bloomed flowers (F3). The GSEA showed that ‘terpenoid backbone biosynthesis’ was only significantly (*p* < 0.05) enriched in F3 vs. F1 (Figure S1). A total of 18 enriched genes were found in F3 vs. F1 and root vs. flower comparisons, most of which were highly expressed in bloomed flowers (Figure 2d). The results suggest that the expression of terpene biosynthesis-related genes is spatiotemporal in *D*. *catenatum* flowers.

### 2.3. Identification of PS-TPSs

**Figure 3:**
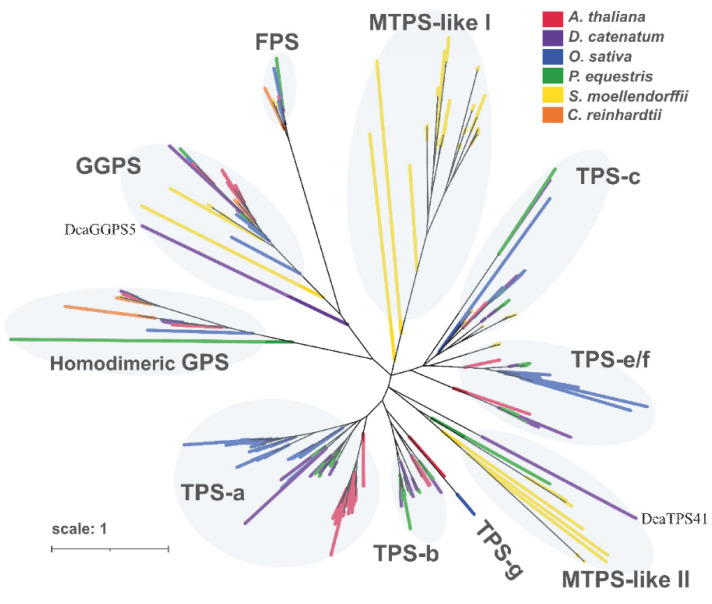
Phylogenetic analysis of PS-TPS proteins in *Arabidopsis thaliana*, *Dendrobium catenatum*, *Oryza sativa*, *Phalaenopsis equestris*, *Selaginella moellendorffi*, and *Chlamydomonas reinhardtii*. A total of 71 PSs and 247 TPSs were used to construct the unrooted maximum likelihood phylogenies.

The precursors of terpenes were modified by PS-TPS, and they played important roles in terpene diversity. Most PS-TPS genes were highly expressed in bloomed flowers (Figure 2d), but the roles of PT-TPS genes remain largely unknown in *D*. *catenatum*. A thorough search of the *D*. *catenatum* genome sequence led to the identification of 9 PSs and 42 TPSs (Table S4). Based on the phylogenetic analysis of PS-TPSs from *D*. *catenatum* and other plants, 71 PSs were divided into three groups (i.e., GGPS, FPS, and GPS), and 247 TPSs were divided into seven groups (i.e., TPS-a, TPS-b, TPS-c, TPS-e/f, TPS-g, TPS-h, and MTPS-like) according to previous studies (Figure 3) [4,6,13,14].

**Figure 4:**
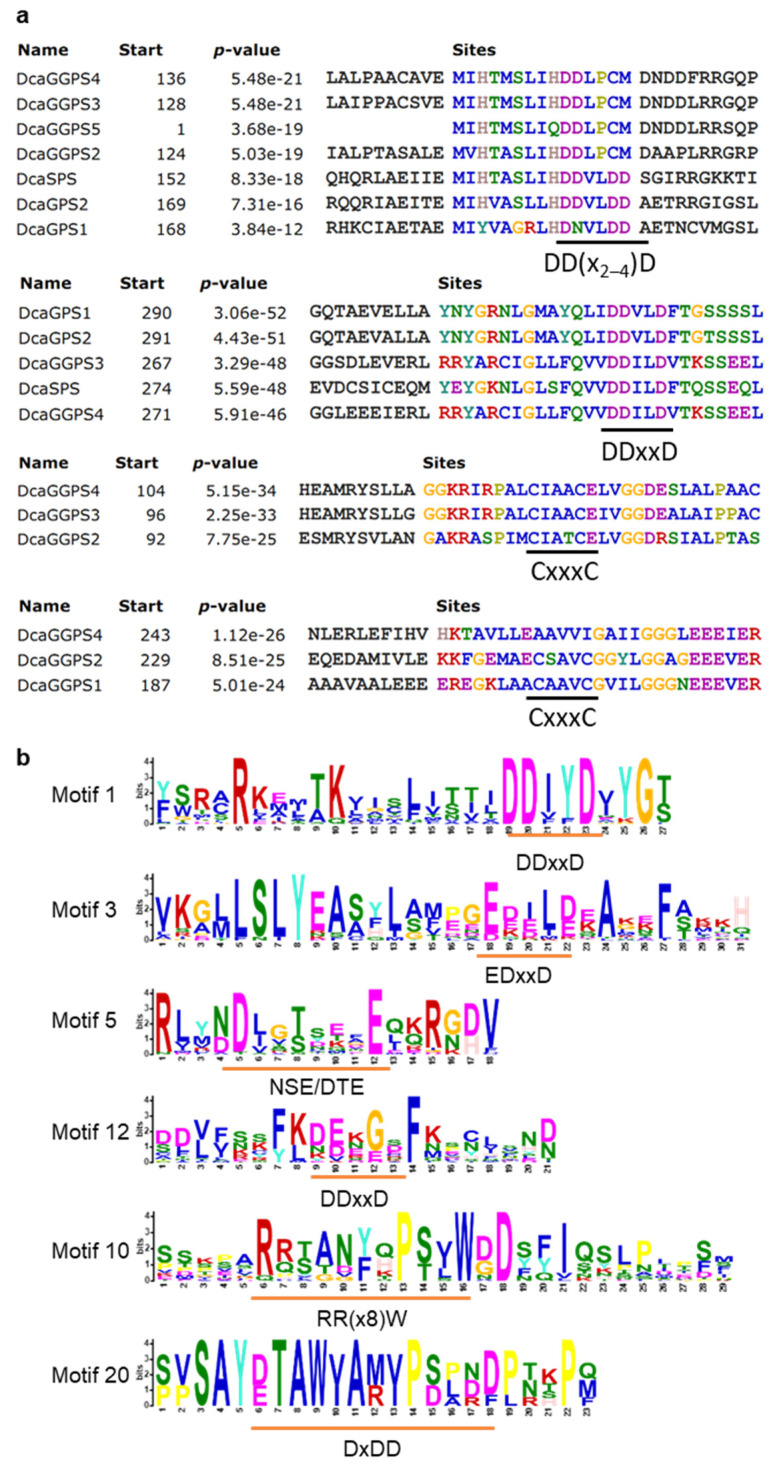
Domain organization of DcaPS-TPS. (**a**) The conserved motifs of DcaPSs. (**b**) The conserved motifs of DcaTPSs. Asp-rich motifs (DDxxD) are found in DcaPS-TPS, where ‘x’ is any amino acid.

The DcaPS family contained five DcaGGPSs, two DcaGPSs, one DcaFPS, and one DcaSPS (Table S4). The aspartate-rich DD(x_2–4_)D (‘x’ is any amino acid) motif and CxxxC motif were found in all DcaPSs, except DcaFPS (Figure 4a). The large subunit of GGPSs (LSU) and GPSs contained the DD(x_2–4_)D motif, while the GGPS small subunit (SSU) lacked this motif and contained the CxxxC motif [14]. However, DcaGGPS2~4 contained both the DD(x_2–4_)D and CxxxC motifs (Figure 4a). Thus, the classification of LUS and SSU according to their motif constitution was not strict in DcaPSs. Another possible reason might be that *D*. *catenatum* genome assembly existed in the gaps and resulted in incomplete sequences.

Furthermore, we searched for 30 conserved motifs in DcaTPSs using MEME software. Most subfamily members showed similar conserved motif distributions (Figure S2). However, several DcaTPSs differed in their homologs, such as DcaTPS09, which might have an incomplete assembly in the genome. Class I activity was found in almost all plant TPSs including the ‘DDxxD’ and ‘NSE/DTE’ motifs [4]. All TPS-a and TPS-b members contained ‘DDxxD’ and ‘NSE/DTE’, except for DcaTPS17. The N-terminal transit peptides of TPS were presumably cleaved off by a ‘RRx8W’ motif [3], which was distributed in the TPS-a and TPS-b subfamilies. The class II activity resided in a separate domain and contained ‘DxDD’ and ‘EDxxD’ motifs [15]. TPSs containing ‘EDxxD’ motifs were members of TPS-d, -c, and e/f [4]. However, ‘EDxxD’ motifs were widely distributed throughout the four DcaTPS subfamilies including TPS-a, TPS-b, TPS-c, and TPS-e/f. Moreover, ‘DDxxD’ and ‘DxDD’ motifs were distributed in all TPS-e/f proteins, and ‘DxDD’ motifs were present in TPS-c with the exception of DoTPS34 (Figure 4b and Figure S2). The genome of *Selaginella moellendorffi* was found to contain two distinct types of TPS genes, typical plant terpene synthases and microbial TPS-like (MTPS-like) synthases [13]. The phylogenetic analysis showed that DcaTPS41 was classified into the MTPS-like II subgroup (Figure 3). The above-mentioned motifs were not found in DcaTPS41 (Figure S2). However, DcaTPS41 contained a conserved ‘terpene_synth_C’ domain (Figure S3). MTPS from *S. moellendorffi* containing only the ‘terpene_synth_C’ domain represented a type of TPS that has been widely identified in bacteria, fungi, and nonseed plants [13]. Thus, DcaTPS41 might be a new kind of MTPS in *D*. *catenatum*.

### 2.4. Spatiotemporal Expression Patterns of DcaPS-TPSs in D. catenatum

**Figure 5:**
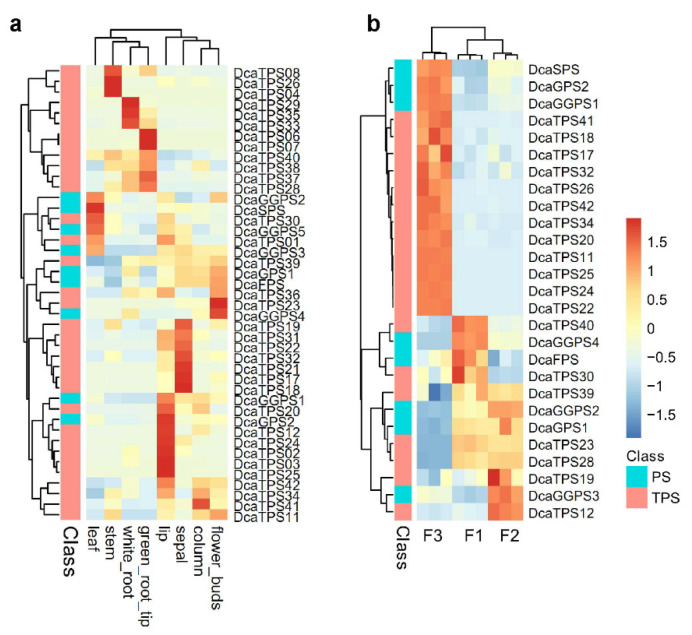
Spatiotemporal expression patterns of *DcaPS-TPS* genes. (**a**) Expression patterns of *DcaPS-TPS* genes in different tissues. (**b**) Expression patterns of *DcaPS-TPS* genes in three flowering phases. In the early developmental stage (F1), the column was short and lip crest were green; in the medium stage of flower bud development (F2), the column and lip had purple pigmentation; when the flower matured and opened (F3), the sepals and petals turned yellow and red pigmentation. Color scale represents the value of the relative expression level log_2_-FPKM.

To investigate the potential roles of *DcaPS*-*TPSs* during growth and development, RNA-seq data from different plant tissues and organs were detected [16]. The *DcaPS*-*TPSs* showed distinct organ-specific expression patterns, and the half of *DcaPS*-*TPSs* were primarily expressed in flowers (Figure 5a). In detail, several genes were highly expressed in specific flower tissues, including *DcaTPS25* and -*03* in lip (labellum), *DcaGGPS4* and *DcaTPS23* in flower bud, and *DcaTPS17* and -*18* in sepal. Furthermore, we explored the expression levels of *DcaPS*-*TPSs* during different flowering phases. *DcaSPS*, *DcaGPS2*, *DcaGGPS1*, and most *DcaTPSs* were highly expressed in F3. The rest of the *DcaPS*-*TPSs* were primarily expressed in F1/F2 (Figure 5b). The results were consistent with the tissue expression profiles of *DcaPS*-*TPSs* and indicated that the organ-specific expression of *DcaPS*-*TPSs* might be important in the terpene biosynthesis of *D. catenatum* flowers.

### 2.5. The Response of DcaPS-TPSs under Abiotic and Biotic Stresses in D. catenatum

**Figure 6:**
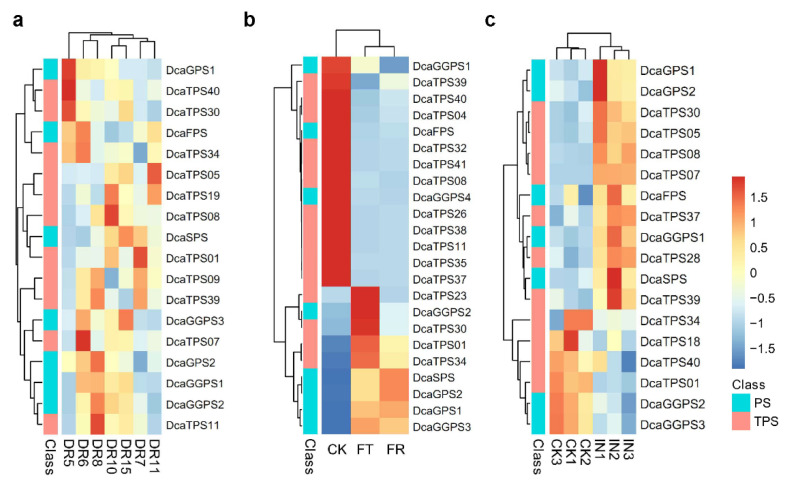
The response of *DcaPS-TPS* genes under abiotic and biotic stresses. (**a**) Expression of *DcaPS-TPS* genes under drought stress. The seedlings were watered on the 1st day, dried from the 2nd to the 7th day, and then rewatered on the 8th day. Leaves were collected at different times; DR5/DR8, DR6/DR10, and DR7/DR15 indicate sampling at 06:30 and 18:30 on the 2nd, 7th, and 9th days, respectively, and DR11 indicates sampling at 18:30 on the 8th day. (**b**) Expression of *DcaPS-TPS* genes under cold stress. Two-year-old cultivated plants were placed at −6 ℃ for freezing treatment (FT) and 8 °C for post freezing-recovery (FR). (**c**) Expression of *DcaPS-TPS* genes during *Colletotrichum gloeosporioides* infection. Two-year-old cultivated plants were infected with *C. gloeosporioides* for 15 days. Color scale represents the value of the relative expression level log_2_-FPKM.

*D. catenatum* is an epiphytic orchid plant that often experiences abiotic and biotic stresses such as drought and low temperatures. We explored the possible roles of *DcaTPSs* in response to drought stress by analyzing the raw RNA-seq reads from the leaves [17]. For drought stress, the plants were irrigated on the first day, dried from the second to the seventh day, and then recovered on the eighth day. Leaves were sampled at dawn (06:30) and dusk (18:30) on the second (i.e., DR5 and DR8), seventh (i.e., DR6 and DR10), and ninth day (i.e., DR7 and DR15), and on the eighth day, DR11 was sampled at 18:30 (Figure 6a). The results showed that rewatering restored the expression levels of *DcaTPS05* and -*19*. For most of the *DcaPS*-*TPSs*, their expression was not always the same under drought stress, such as for *DcaGPS1*; *DcaGGPS2*; *DcaTPS11*, -*39*, and -*40*, which were induced on DR5 and repressed on DR8. This suggests that these *DcaPS*-*TPSs* might be regulated by the biological clock under drought stress.

We further identified *DcaPS*-*TPS* expression profiles under control (CK), freezing treatment (FT), and post-freezing recovery (FR). Fourteen *DcaPS*-*TPSs* were downregulated, and six *DcaPS*-*TPSs* were significantly upregulated under cold stress. The expressions of three *DcaPS*-*TPSs* were only induced under FT including *DcaGGPS2* and *DcaTPS23* and -*30* (Figure 6b). These results indicate that several *DcaTPSs* were transcriptionally responsive to cold stress.

To understand the role of *DcaPS*-*TPSs* in disease resistance, *D. catenatum* leaves were inoculated with *Colletotrichum gloeosporioides*. The expression profiles of *DcaPS*-*TPSs* showed that 12 *DcaPS*-*TPSs* were upregulated and 6 *DcaPS*-*TPSs* were downregulated in infected leaves (Figure 6c). A Venn diagram shows that 13 *DcaPS*-*TPSs* were identified in three transcriptome data, and most of them were detected in four tissues (Figure S4). These results suggest that *DcaPS*-*TPSs* might be involved in enhancing defense ability under abiotic and biotic stresses.

### 2.6. Cis-Elements in the Promoter Regions of DcaPS-TPSs

**Figure 7:**
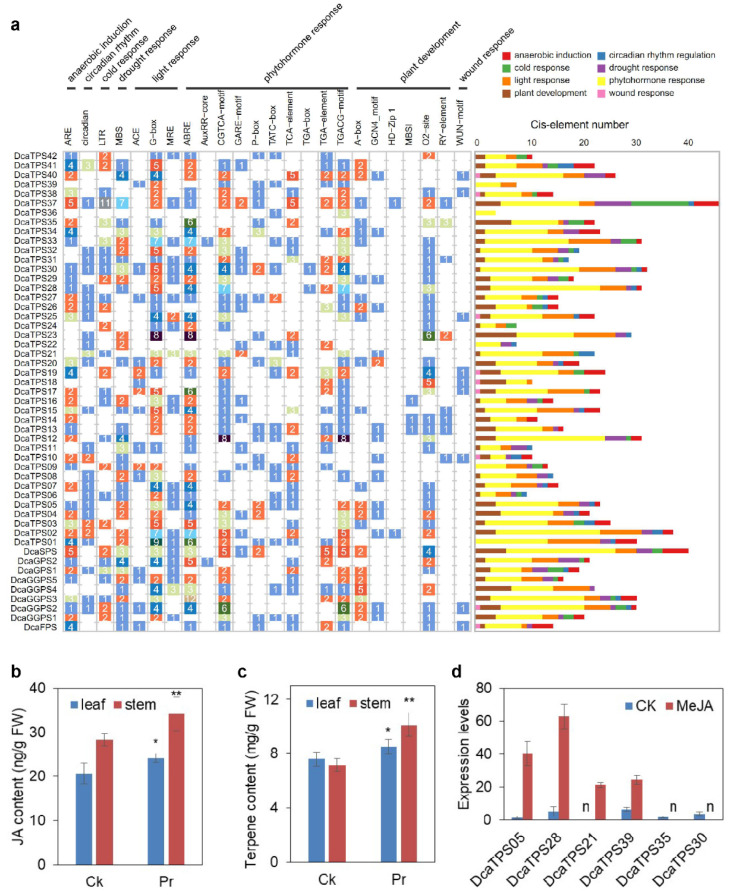
Predicted cis-elements in *DcaPS-TPS* promoters and the response of *DcaPS-TPS* under MeJA treatment. (**a**) Promoter sequences (−2000 bp) of *DcaPS-TPS* genes were analyzed by PlantCARE. The classification and number of cis elements were shown on top and left, respectively. (**b**) The endogenous JA content of in Pr and control plants. Values are means ± S.D. (*n* = 6). Student’s *t*-test, * *p* < 0.05, ** *p* < 0.01. (**c**) The total terpene content of in Pr and control plants. Values are means ± S.D. (*n* = 6). Student’s *t*-test, * *p* < 0.05, ** *p* < 0.01. (**d**) The expression levels of six *DcaPS-TPS* genes under MeJA treatment. Values are means ± S.D. (*n* = 3). n, not detectable.

To predict the putative functions of *DcaPS*-*TPSs* in response to biotic and abiotic stresses, we analyzed 2 kb upstream of the *DcaPS*-*TPSs* (Figure 7a). Potential cis-elements of the *DcaPS*-*TPS* promoter were determined using PlantCARE. Twenty-four regulatory sequences were found in the promoters of *DcaPS-TPSs* including abiotic stress-responsive cis-elements (e.g., MBS in the drought response and LTR in cold stress), plant growth and development-related cis-elements (e.g., circadian, A-box, and O2-site), and phytohormone-related cis-elements (e.g., the GARE-motif of the gibberellin response, ABRE of the abscisic acid response, and the TCA-element of the salicylic acid response). Most of the *DcaPS-TPS* promoters contained light-responsive elements, suggesting that *DcaPS-TPSs* might play important roles in plant photoperiod regulation.

As plant-specific signaling molecules, jasmonates (JAs) not only steer the response of plant stress but also induce the accumulation of secondary metabolite production [18,19]. Most of the *DcaPS-TPS* promoters contained CGTCA-motifs of JA response (Figure 7). Our previous study reported a purple (Pr) variety of *D*. *catenatum* that enriched more flavonoids in stems [12]. The endogenous JA and total terpene contents were determined in Pr and control plants. Significant increases in the JA and terpene content were observed in Pr (Figure 7b,c). Furthermore, the expression levels of 35 *DcaPS-TPSs* were detected in Pr and CK by transcriptome analysis. Six *DcaPS-TPSs* were upregulated and ten *DcaPS-TPSs* were downregulated (fold change > 2) in Pr vs. CK (Figure S5). We selected six *DcaPS-TPSs* to detect the exogenous MeJA effect in *D*. *catenatum* stems. *DcaTPS05*, -*21*, -*28*, and -*39* were induced under MeJA treatment and *DcaTPS30* and -*35* were suppressed under MeJA treatment (Figure 7d). These results suggest that JA is involved in the regulation of *DcaTPS* expression and terpene biosynthesis.

## 3. Discussion

As it is a valuable traditional herb in China, the extraction of *D*. *catenatum* displays strong immune modulatory activity, and is used for relieving stomach upsets and its antitumor and antipyresis properties [10,20]. To date, research on the secondary metabolites in *D*. *catenatum* primarily focuses on phenylpropanoid biosynthesis such as flavonol and anthocyanin [12,21]. MeJA treatment and mycorrhizal fungi infection could induce dendrobine biosynthesis in *Dendrobium* [11,22]. Dendrobine, a sesquiterpene alkaloid, is regarded as the standard of quality for *Dendrobium* stem [10]. However, no reports have been published on the distributions of terpenes in *D*. *catenatum*. In this study, 41 volatile terpenes and 33 non-volatile terpenes were detected (Figure 1d). Most of them were first reported in *D*. *catenatum*. For instance, isolongifolene, which is more highly accumulated in the root (Figure 1g), has been previously demonstrated to have a neuroprotective effect against Parkinson’s disease [23]. Bisabolene, epizonarene, and muurolene primarily accumulate in the stem (Figure 1h), but their function is still unknown. Interestingly, we found that most of the terpene biosynthesis-related genes were positively correlated with tissue-specific accumulation of terpenes, such as monoterpenes, primarily in the flowers (Figure S7). *Dendrobium* flower has been used as a health care tea for its light scent and medicinal value, but its aroma composition is still unclear. Myrcene, ocimene, α-pinene, and α-terpineol are considered to be the major floral fragrance compositions [24]. These monoterpenes were also detected in *D*. *catenatum* (Table S1). Moreover, we found two terpene derivatives, 6-hydroxydendroxine and α-amyrenone, which were only detected in *D*. *catenatum* flowers (Figure 1h). 6-Hydroxydendroxine and α-amyrenone have an important medicinal value. 6-Hydroxydendroxine is a derivative of dendroxine, and it is especially common in orchidaceae [25]. Recently, α-amyrenone has been found to have anti-hyperglycemic, lipid-lowering, and anti-obesity effects [26]. Thus, the medicinal value of *D*. *catenatum* is yet to be discovered.

Previous research has focused on the putative upstream elements of the alkaloid biosynthetic pathway, which overlap with the terpene biosynthesis pathway [11]. Terpenes originated via de novo synthesis from IPP and DMAPP, and they were further catalyzed to produce short-chain products by PSs early in the isoprenoid pathway [27]. PSs belong to the chain elongation family (PF00348) within the terpene synthase superfamily, whose members also include squalene/phytoene synthase and TPS [28]. All proteins in the terpene synthase superfamily contain a characteristic all α-helix motif [28]. However, none of the previous studies grouped PS and TPS together in Orchidacea [29]. In this study, DcaPT-TPSs contained five conserved regions within a six-helix bundle that contained aspartate-rich DD(x_2–4_)D pyrophosphate-binding motifs (Figure 4). However, the conserved motifs of the DcaFPS protein were not found with other DcaPTs by MEME motif analysis. We further aligned the sequence of DcaFPS with AtFPS1 and -2, which all contained aspartate-rich DDxxD motifs (Figure S6). Phylogenetic analysis showed that DcaFPS is grouped with FPSs of Arabidopsis and rice (Figure 3). This suggests that FPSs are evolutionarily conserved in dicots and monocots. Moreover, previous studies reported 34 TPSs in *D. officinale* and 35 TPSs in *D*. *catenatum* [29,30]. In this study, 42 TPS genes were found in the *D*. *catenatum* genome v.2.2 (Table S4). Genome v.2.2 (NCBI_Assembly:GCA_001605985.2) sequences are not complete and many gaps exist [31]. This is probably why several TPS motifs were not detected in the MEME analysis (Figure S2). These TPSs were divided into four subfamilies (Figure S2), which is in accordance with previous studies [29], except for DcaTPS41. TPSs have additional β and/or γ domains in plants, not only an α motif [4,13,28]. However, these motifs were not found in DcaTPS41 (Figure S2). Phylogenetic analysis showed that DcaTPS41 and its *P. equestris* homolog were more closely related to SmMTPS (Figure 3). Plant MTPSs were first found in *S*. *moellendorfi* and are regarded as a special group in nonseed plants [13], but the sequence alignment showed that *Phytolacca bogotensis* and *Opuntia* sp. contained one and four MTPSs, respectively [32]. Orchidaceae seed is well known for its symbiotic germination with mycorrhizal fungi [33]. *Dendrobium* seeds will not germinate without the fungus to feed them [34]. However, no studies have focused on the symbiotic relationship between *Dendrobium* and fungus in vivo. We believe that high-quality genome assembly and omics analysis will facilitate MTPS study in *Dendrobium*.

*D*. *catenatum* is an epiphytic plant that grows in a warm and humid environment through its special aerial roots. Aerial root development is associated with a high degree of IAA accumulation and is promoted by the light signaling pathway [35]. Some *AtTPSs* exhibited root zone-specific expression patterns in Arabidopsis, such as the duplicated genes *AtTPS23* and *AtTPS27*, which produced the monoterpene 1,8-cineole and were expressed in the stele and epidermis of the root [4]. *AtTPS08* encoding a putative diterpene synthase was expressed in the stele, and *AtTPS22* encoding a putative sesquiterpene synthase was expressed in the lateral root cap [36]. Interestingly, we found that three *DcaPS-TPSs* were expressed specifically in green root tips, and two were especially expressed in white root (Figure 5a). This could be associated with root growth and development. Moreover, monoterpenes are major volatile constituents of many plants, and they are catalyzed by TPS [24]. The GSEA indicated that terpene biosynthesis-related genes were mainly expressed in floral organs (Figure 2a). Recently, one *TPS* gene from *D. officinale* showed the highest transcript level in floral organs and could only covert GPP to linalool in vitro [30]. Linalool is one of the predominant components of floral scents [24], but GC-MS did not detect it in the flower (Table S1). This suggests that terpene biosynthesis regulation is more complex in vivo.

Terpenes play significant ecological roles in the interactions between plants and environmental stresses. As the core genes in terpene biosynthesis, the expression levels of *PS-TPS* genes in different plants under various stress conditions have been widely reported. Five strawberry *TPSs* were significantly upregulated in response to anthracnose at 3–6 h post-infection [37]. *TPS21* encoding sesquiterpene synthase was negatively correlated with dendrobine accumulation after infection with mycorrhizal fungus in *D*. *nobile* [22]. Both the *AtTPS03* and *AtTPS04* genes were induced locally at wound sites to promote biosynthesis of volatile terpenes [9,38]. In addition, terpene metabolism is related to phytohormone biosynthesis, such that gibberellins (GAs) are tetracyclic diterpenoids that are biosynthesized from GGPP. Except for GA_20_, GA_7_, and GA_8_, which increased in Pr, the others were all reduced in Pr (Figure S8). Moreover, the endogenous JA and total terpene levels increased in Pr (Figure 7b,c). Not all *DcaTPS* genes were induced by JA treatment (Figure 7d). These results suggest that terpene biosynthesis was induced by JA crosstalk with GA. The regulation of *DcaTPSs* expression was influenced by multiple factors, and the functionality of *DcaTPSs* needs to be studied further.

## 4. Materials and Methods

### 4.1. Identification of PS-TPSs Family

The PS-TPS sequences including genomic DNA and proteins in *Arabidopsis thaliana*, *Oryza sativa*, *Phalaenopsis equestris*, *Selaginella moellendorffi*, and *Chlamydomonas reinhardtii* were retrieved from Phytozome v12 (https://phytozome.jgi.doe.gov/pz/portal.html, accessed on 23 March 2021). The genome of *Dendrobium catenatum* was downloaded from NCBI (https://www.ncbi.nlm.nih.gov/genome, accessed on 4 May 2020). The PS-TPS proteins were originally obtained using the hidden markov model (HMM) search and BLASTP search. Three domains, PF03936, PF19086 and PF01397 were used to search for TPSs, and PF00348 was used to search for PSs. The significant hits were selected with an *E* value < 1 × 10 ^−10^ as the cutoff. All the hits were further confirmed on NCBI-CDD (https://www.ncbi.nlm.nih.gov/cdd, accessed on 27 March 2021) and SMART (http://smart.embl-heidelberg.de/, accessed on 27 March 2021).

### 4.2. Analysis of Phylogenetic Relationship, Motif Architecture, and Cis-Elments of Promoters

The sequences of PS-TPS proteins from these species were aligned with ClustalW software and then an un-rooted tree was constructed using FastTree with options by default [39,40]. The motif analysis was performed in MEME (https://meme-suite.org/meme/, accessed on 2 April 2021). The cis-elements of *DcaPS-TPSs* promoters were analysed in PlantCARE (http://bioinformatics.psb.ugent.be/webtools/plantcare/html/, accessed on 2 April 2021).

### 4.3. In Silico Expression Profiling of DcaPS-TPSs

For expression profiling of *DcaPS-TPSs*, the raw RNA-seq data of tissues (PRJNA348403 and PRJNA680456) were downloaded from the NCBI [16,41]. For drought stress, 8-month-old seedlings were transferred into an incubator (12/12 h light/dark, light intensity 100 μmol m^−2^ s^−1^; 28/22 °C day/night; and relative humidity 60/70% day/night) and adapted to the controlled conditions for 10 days. Plants were irrigated on the 1st day, dried from the 2nd to the 7th day, and then resumed on the 8th day. The raw RNA-seq data of drought stress were obtained from Sci Data [17]. The RNA-seq and metabolome data of Pr were described in our previous studies [12]. For fungus treatments, two-year-old cultivated plants were infected with *C. gloeosporioides* for 25 d in greenhouse (natural condition in April 2021, Zhejiang University, Hangzhou, China). The raw RNA-seq data of *C. gloeosporioides* infected plants were submitted to the BIG Data Center of the Chinese Academy of Sciences (https://bigd.big.ac.cn (accessed on 6 May 2022)) with accession number CRA002691. For cold stress, two-year-old cultivated plants were placed at 0 °C and then subjected to a gradual drop from 0 to −6 °C within 3 h. The temperature was held at −6 °C for 3 h and then held at 8 °C for 24 h without lighting. The raw RNA-seq data of cold stress have been submitted to the BIG Data Center with accession number CRA003229 and CRA005177 [42]. The quality control of raw data and mapping of reference genomes were performed according to previous work [12]. In brief, the raw data were first qualified using trimmomatic (v.0.39, accessed on 6 July 2021) with the parameter ‘ILLUMINACLIP: TruSeq3-PE-2′ to obtain clean data. Clean data were aligned to the reference genome using hisat2 (v.2.2.1, accessed on 7 July 2021) and then were counted using featureCount (v.2.0.3, accessed on 7 July 2021) to obtain the expression matrices with the default parameters. Subsequently, the expression levels of unigene were calculated as FPKM (fragments per kilo bases of exons for per million mapped reads). *DcaPS-TPSs* expression matrices were treated by hierarchical clustering using the R package ggplot2 (v.3.3.5, accessed on 7 July 2021).

### 4.4. MeJA Treatment and Real-Time Quantitative PCR

Two-year-old cultivated *D*. *catenatum* were grown at 20 ± 2 °C with a light/dark cycle of 12/12 h and a 65–75% relative humidity as a control treatment. For MeJA treatment, the leaves were sprayed with 1 mM MeJA for 24 h. The leaves were harvested and immediately frozen in liquid nitrogen.

Total RNA was isolated from leaf samples using TransZol reagent (TransGen Biotech, Beijing, China). First-strand cDNA was reverse transcribed using the TIANscript RT Kit according to the manufacturer’s instructions (TransGen Biotech, Beijing, China). Quantitative real-time PCR analyses were performed using a SYBRGreen qPCR kit (TransGenBiotech) with a MyiQ system (Bio-Rad, Hercules, CA, USA) as described previously [43]. The primers for amplification are listed in Table S6.

### 4.5. Metabolomics and Analysis

Two-year-old cultivated *D. catenatum* were grown in greenhouse (natural condition in May 2021, Zhejiang University, Hangzhou, China). The *D. catenatum* flowers were in full bloom in May. The tissue samples were collected from six independent plants (three biological replicates) and immediately ground into powder in liquid nitrogen.

For volatile organic compounds analysis, 1 g powder was transferred to an SPME vial containing 10 μL heptanone as an internal standard. A preconditioned SPME fiber (120 µm DVB/CAR/PDMS, Agilent, Santa Clara, CA, USA) was then exposed to the headspace of the capped vial at 100 °C for 15 min. The fiber coating was carried out in the injection chamber of the GC (Agilent 8890) in splitless mode at 250 °C for 5 min. Quantification of volatiles was carried out using GC (Agilent 8890) and mass spectrometer (Agilent 5977B), and equipped with a capillary DB-5MS column (30 m × 0.25 mm inner diameter with 0.25 μm film thickness). Helium was used as the carrier gas at a linear velocity of 1.0 mL/min. The injector temperature was kept at 250 °C and the detector at 280 °C. The GC oven temperature was programmed at 40 °C for 2min, increased from 40 to 280 °C at 7 °C/min, and then held for 5 min. The mass spectra were taken at electron ionization at 70 eV, and the mass range was 50–500 *m*/*z*. The quadrupole mass detector, ion source and transfer line temperatures were set at 150, 230, 280 °C, respectively. Compounds were identified by matching the data from the MWGC library.

For non-volatile organic compounds analysis, 100 mg powder was dissolved in 1.2 mL 70% methanol solution at 4 °C overnight. UPLC-ESI-MS/MS conditions were performed according to our previous work [12]. The metabolite counts were treated by hierarchical clustering using the R package ggplot2 (v.3.3.5).

## Data Availability

All data generated or analyzed during this study are included in this published article and its Additional files. The datasets generated and analyzed during the current study are available from the corresponding author on reasonable request.

## Supplementary Materials

The following supporting information can be downloaded at: https://www.mdpi.com/article/10.3390/ijms23126398/s1.
