# Supplementary material for: Metabolic Profiling of Terpene Diversity and the Response of Prenylsynthase-Terpene Synthase Genes during Biotic and Abiotic Stresses in Dendrobium catenatum
Source: Int J Mol Sci. 2022 Jun 7;23(12):6398. doi: 10.3390/ijms23126398 (PMC9223610; doi:10.3390/ijms23126398)
Supplement: Supplementary file 1 [file ijms-23-06398-s001.zip › ijms-1758538-supplementary/Figure-suppl.pptx]

## Slide 1
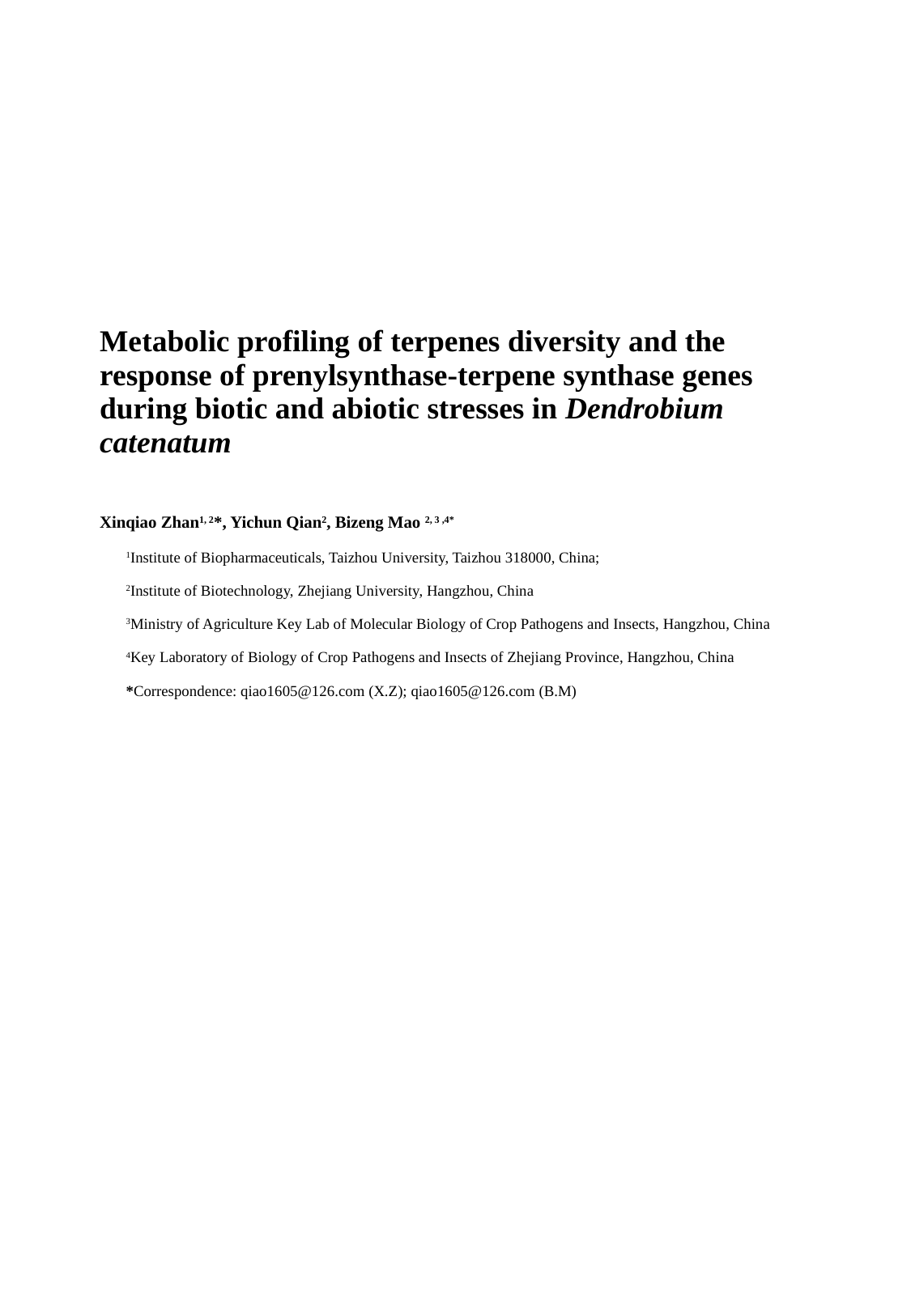

| Metabolic profiling of terpenes diversity and the response of prenylsynthase-terpene synthase genes during biotic and abiotic stresses in Dendrobium catenatum |
| --- |
| Xinqiao Zhan1, 2\*, Yichun Qian2, Bizeng Mao 2, 3 ,4\* |
| 1Institute of Biopharmaceuticals, Taizhou University, Taizhou 318000, China; |
| 2Institute of Biotechnology, Zhejiang University, Hangzhou, China |
| 3Ministry of Agriculture Key Lab of Molecular Biology of Crop Pathogens and Insects, Hangzhou, China |
| 4Key Laboratory of Biology of Crop Pathogens and Insects of Zhejiang Province, Hangzhou, China |
| \*Correspondence: qiao1605@126.com (X.Z); qiao1605@126.com (B.M) |

## Slide 2
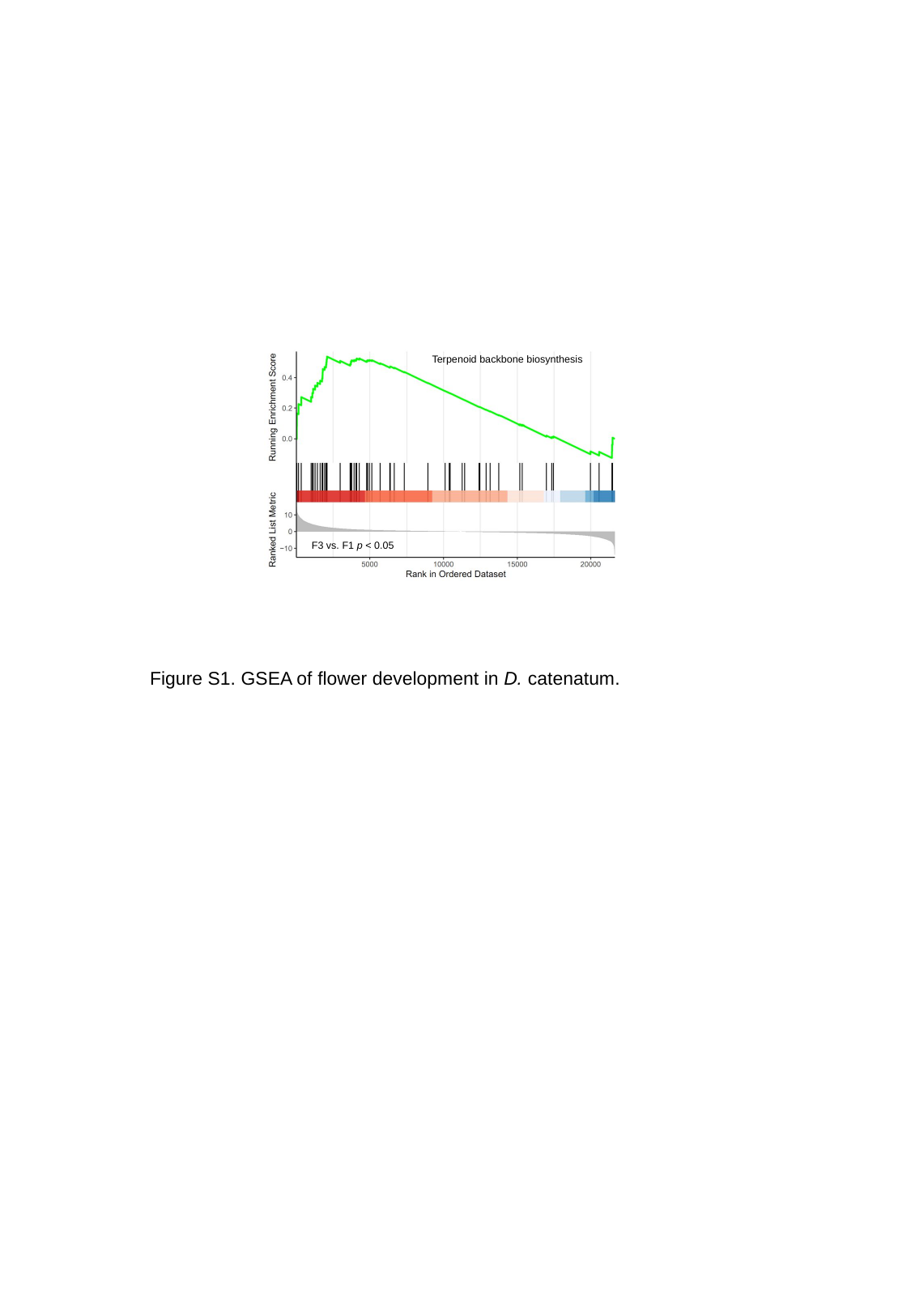

Terpenoid backbone biosynthesis
F3 vs. F1 p < 0.05
Figure S1. GSEA of flower development in D. catenatum.

## Slide 3
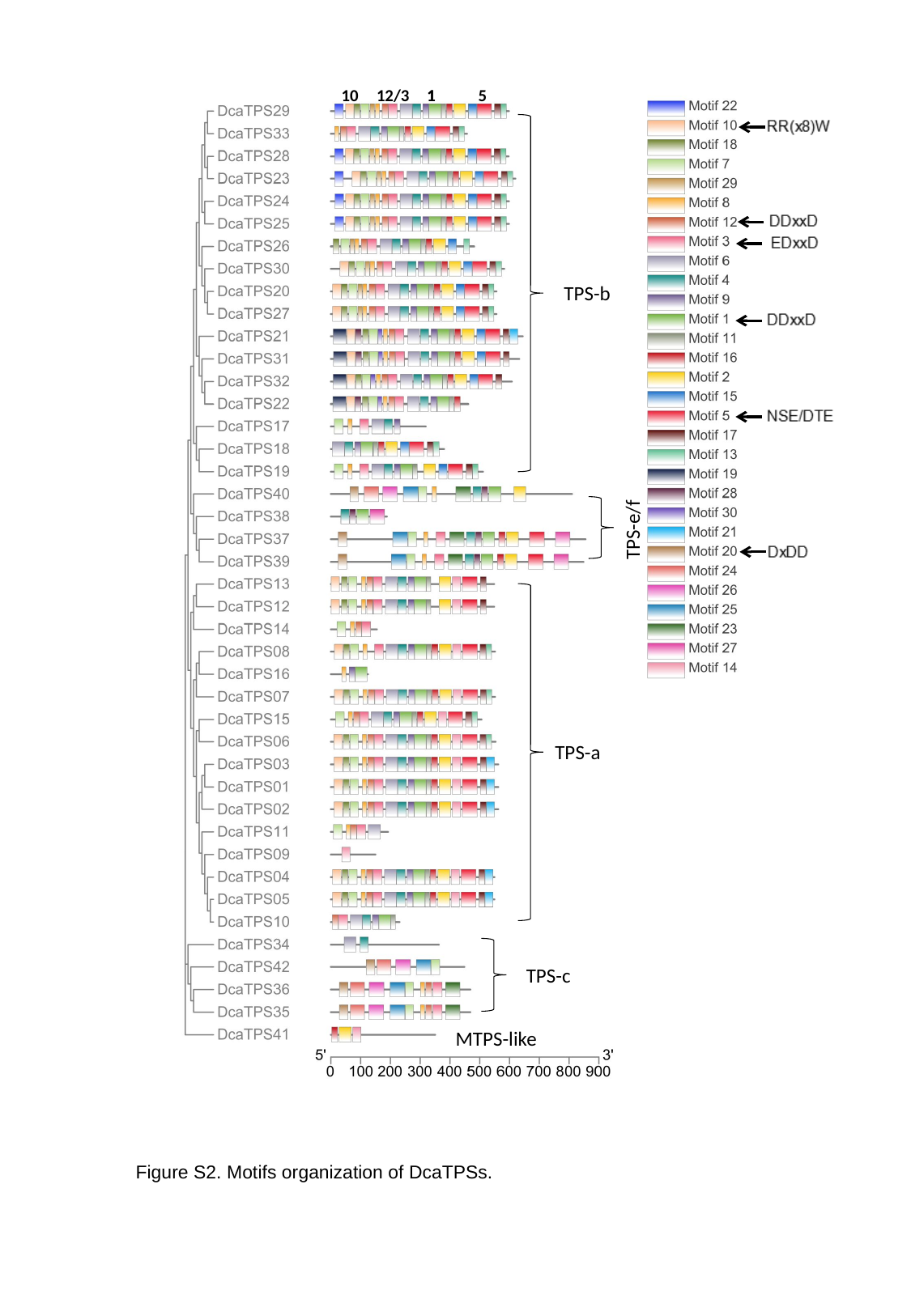

10
12/3
1
1
5
TPS-b
TPS-e/f
TPS-a
TPS-c
MTPS-like
Figure S2. Motifs organization of DcaTPSs.

## Slide 4
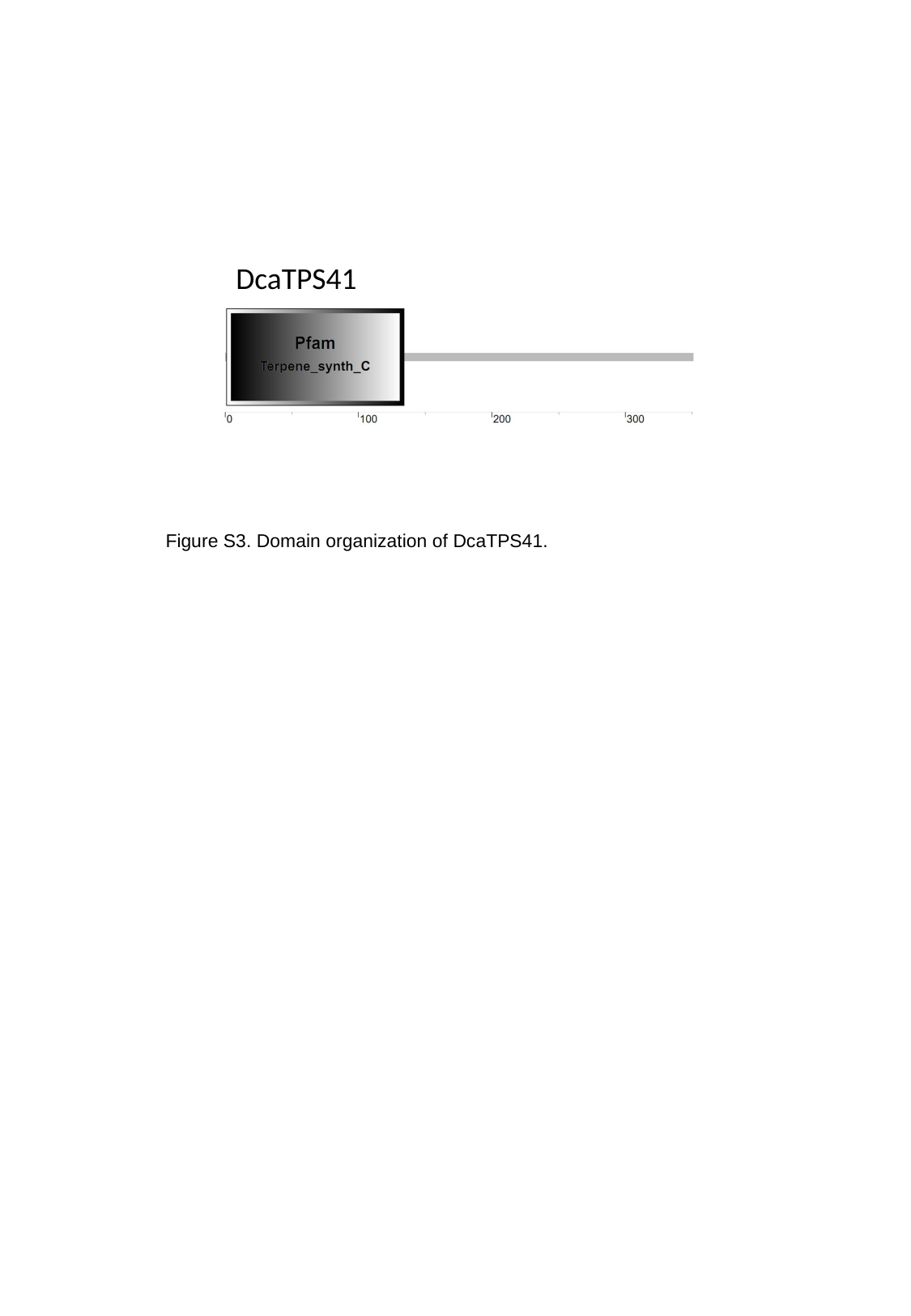

DcaTPS41
Figure S3. Domain organization of DcaTPS41.

## Slide 5
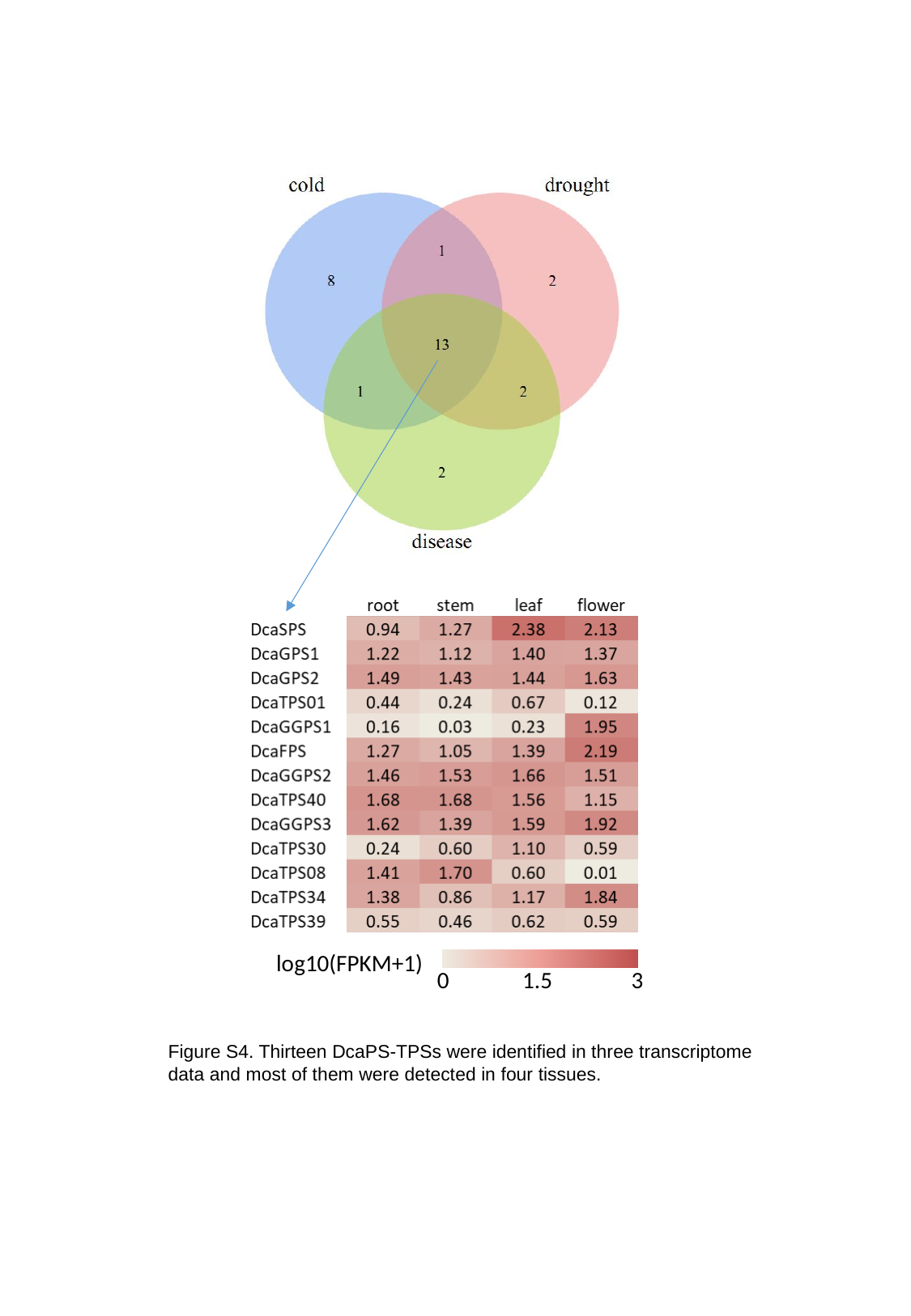

log10(FPKM+1)
0 1.5 3
Figure S4. Thirteen DcaPS-TPSs were identified in three transcriptome data and most of them were detected in four tissues.

## Slide 6
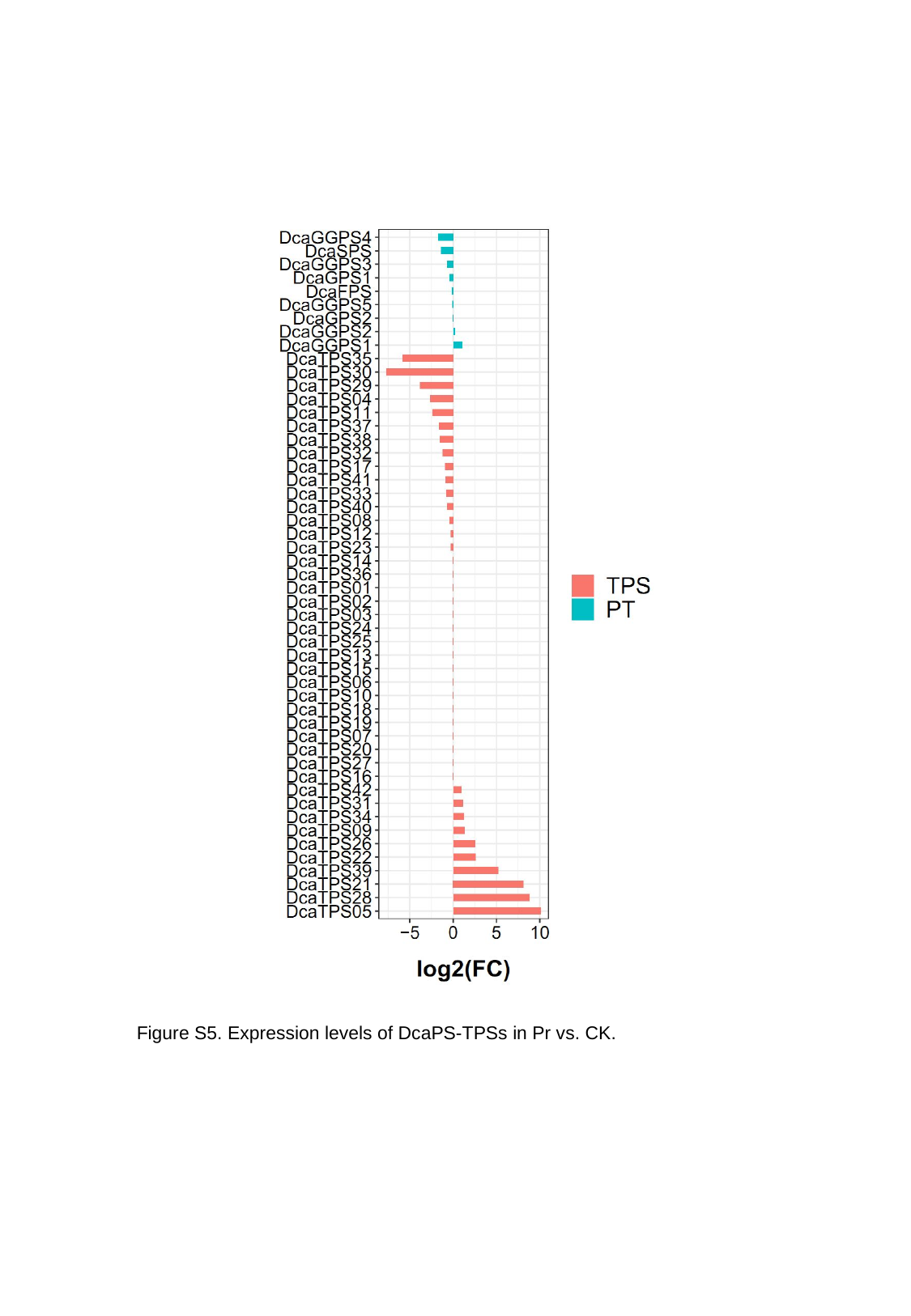

Figure S5. Expression levels of DcaPS-TPSs in Pr vs. CK.

## Slide 7
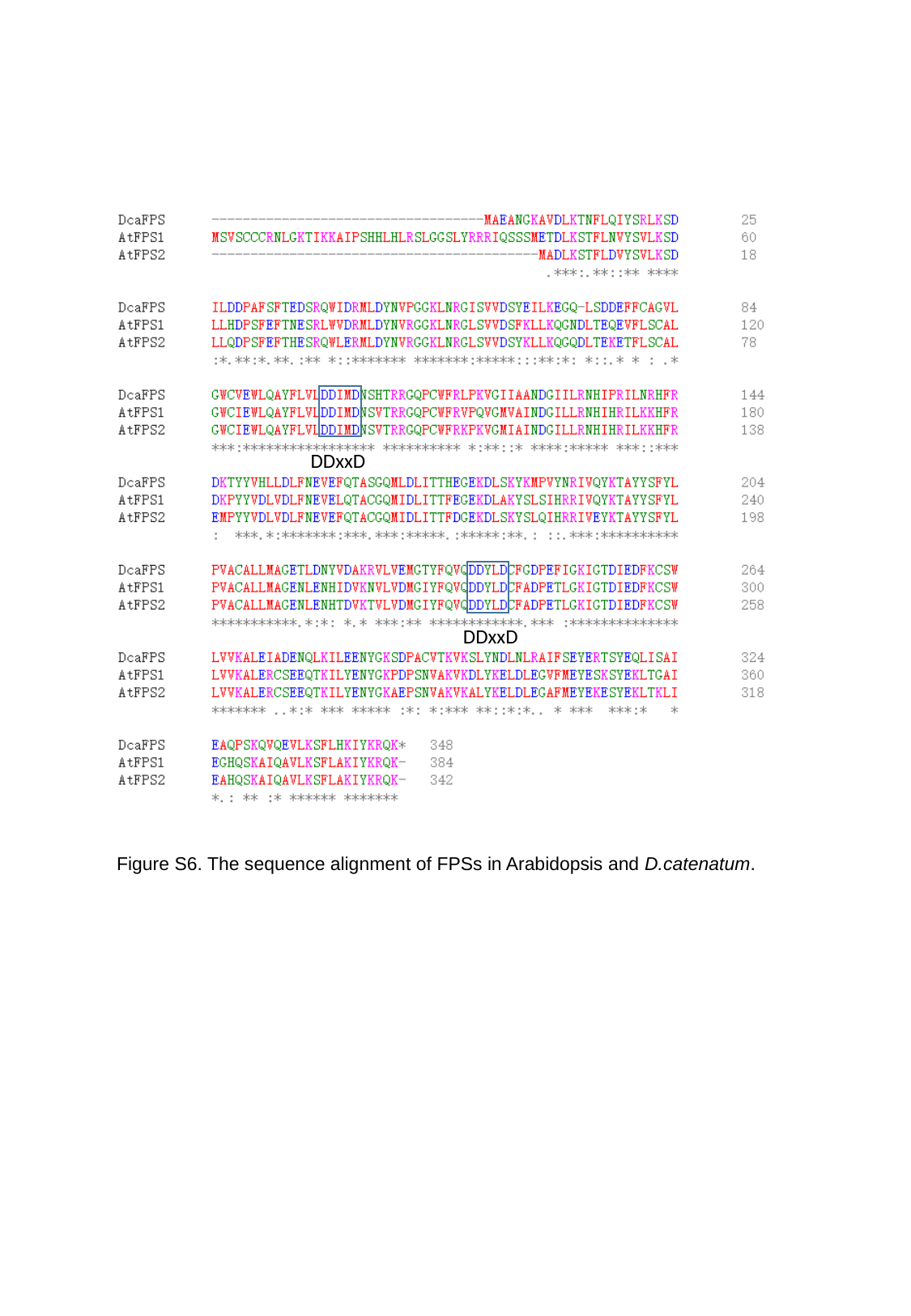

DDxxD
DDxxD
Figure S6. The sequence alignment of FPSs in Arabidopsis and D.catenatum.

## Slide 8
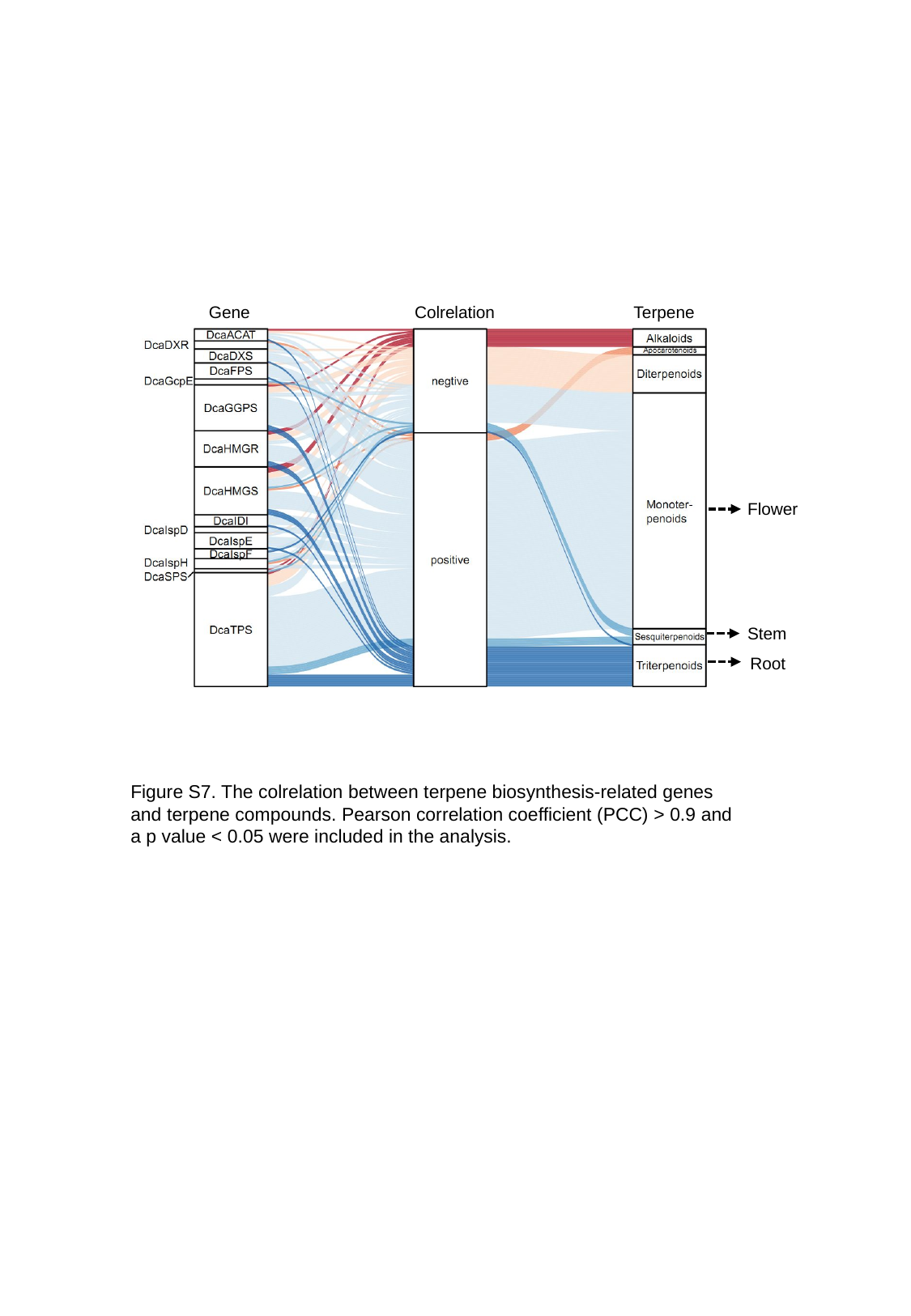

Colrelation
Terpene
Gene
Flower
Stem
Root
Figure S7. The colrelation between terpene biosynthesis-related genes and terpene compounds. Pearson correlation coefficient (PCC) > 0.9 and a p value < 0.05 were included in the analysis.

## Slide 9
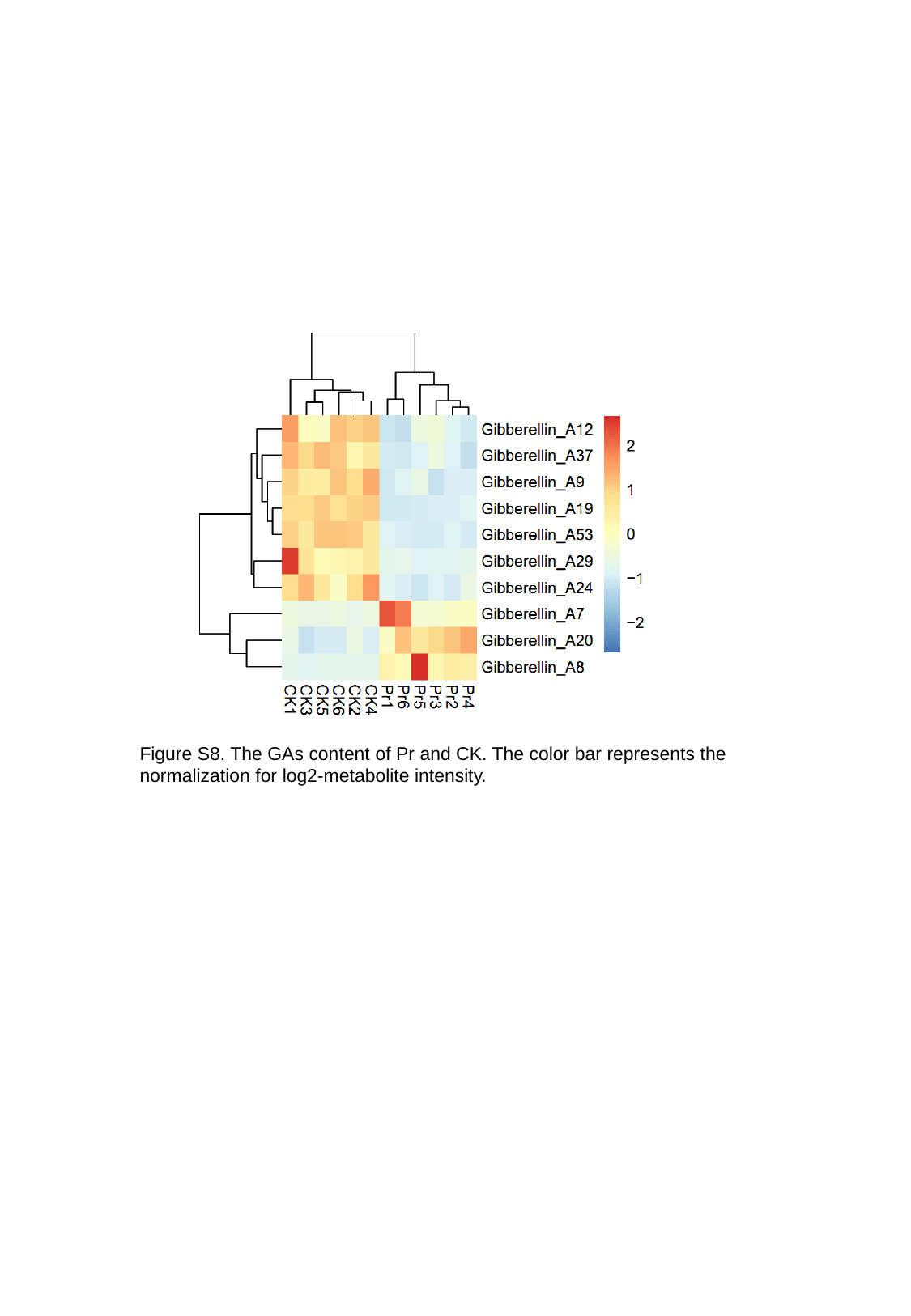

Figure S8. The GAs content of Pr and CK. The color bar represents the normalization for log2-metabolite intensity.
